# Integrating network pharmacology, molecular docking and non-targeted serum metabolomics to illustrate pharmacodynamic ingredients and pharmacologic mechanism of Haizao Yuhu Decoction in treating hyperthyroidism

**DOI:** 10.3389/fendo.2024.1438821

**Published:** 2024-09-25

**Authors:** Wenbin Huang, Xiaoju Liu, Xingjia Li, Ruixiang Zhang, Guofang Chen, Xiaodong Mao, Shuhang Xu, Chao Liu

**Affiliations:** ^1^ Endocrine and Diabetes Center, The Affiliated Hospital of Integrated Traditional Chinese and Western Medicine, Nanjing University of Chinese Medicine, Nanjing, China; ^2^ Key Laboratory of Traditional Chinese Medicine (TCM) Syndrome and Treatment of Yingbing (Thyroid Disease) of State Administration of Traditional Chinese Medicine, Jiangsu Province Academy of Traditional Chinese Medicine, Nanjing, China

**Keywords:** Haizao Yuhu Decoction, hyperthyroidism, network pharmacology, molecular docking, non-targeted serum metabolomics

## Abstract

**Objective:**

To explore the pharmacodynamic ingredients and pharmacologic mechanism of Haizao Yuhu Decoction (HYD) in treating hyperthyroidism via an analysis integrating network pharmacology, molecular docking, and non-targeted serum metabolomics.

**Methods:**

Therapeutic targets of hyperthyroidism were searched through multi-array analyses in the Gene Expression Omnibus (GEO) database. Hub genes were subjected to the construction of a protein-protein interaction (PPI) network, and GO and KEGG enrichment analyses. Targets of active pharmaceutical ingredients (APIs) in HYD and those of hyperthyroidism were intersected to yield hub genes, followed by validations via molecular docking and non-targeted serum metabolomics.

**Results:**

112 hub genes were identified by intersecting APIs of HYD and therapeutic targets of hyperthyroidism. Using ultra-high performance liquid chromatography with quadrupole time-of-flight mass spectrometry (UPLC-Q-TOF-MS) in both negative and positive ion polarity modes, 279 compounds of HYD absorbed in the plasma were fingerprinted. Through summarizing data yielded from network pharmacology and non-targeted serum metabolomics, 214 common targets were identified from compounds of HYD absorbed in the plasma and therapeutic targets of hyperthyroidism, including PTPN11, PIK3CD, EGFR, HRAS, PIK3CA, AKT1, SRC, PIK3CB, and PIK3R1. They were mainly enriched in the biological processes of positive regulation of gene expression, positive regulation of MAPK cascade, signal transduction, protein phosphorylation, negative regulation of apoptotic process, positive regulation of protein kinase B signaling and positive regulation of MAP kinase activity; and molecular functions of identical protein binding, protein serine/threonine/tyrosine kinase activity, protein kinase activity, RNA polymerase II transcription factor activity, ligand-activated sequence-specific DNA binding and protein binding. A total of 185 signaling pathways enriched in the 214 common targets were associated with cell proliferation and angiogenesis.

**Conclusion:**

HYD exerts a pharmacological effect on hyperthyroidism via inhibiting pathological angiogenesis in the thyroid and rebalancing immunity.

## Introduction

Hyperthyroidism manifests as the excitation of multiple systems and accelerated metabolism due to increased thyroid hormones in the circulatory system (1). Graves’ disease (GD) is the most common cause of hyperthyroidism. An epidemiological survey from 31 provinces of the Chinese mainland reported that the prevalence of clinical hyperthyroidism and GD is 0.78% and 0.53%, respectively (2). Antithyroid drugs (ATDs), radioactive iodine therapy, and surgery are currently optional for hyperthyroidism, although the latter two may cause hypothyroidism. ATDs serve as the first-line treatment of hyperthyroidism (1). Regular monitoring of thyroid function is essential during the long-term treatment of ATDs, and some hyperthyroidism patients may suffer from adverse drug events (ADEs) like leukopenia, liver dysfunction, and vasculitis. A high recurrent rate of hyperthyroidism after withdrawal of ATDs should be considered as well, which is inflated to 53.7% in our previous study (3, 4).

The therapeutic effect of iodine-enriched traditional Chinese herbal medicines on hyperthyroidism has been increasingly recognized. Haizao Yuhu Decoction (HYD) is a canonical iodine-enriched traditional Chinese medicine (TCM) prescription for treating thyroid diseases, including goiter, hyperthyroidism, Hashimoto’s thyroiditis, and thyroid cancer. In a cohort involving 82 women of childbearing age with hyperthyroidism, a 3-month intervention of HYD provides a higher overall efficacy and a lower recurrence rate than methimazole (5). *The Consensus on the Treatment of Graves’s Disease with Iodine-rich Chinese Medicine* recommends the use of iodine-rich traditional Chinese herbal medicines for hyperthyroidism patients who are intolerant to ATDs and refusal of 131iodine therapy or surgery (6).

HYD was first described in Waike Zhengzong (7). It contains 12 traditional Chinese herbal medicines of the *Sargassum pallidum (Turn.) C. Ag* (Hai Zao), *Laminaria japonica Aresch* (Hai Dai), *Laminaria japonica Aresch* (Kun Bu), *Citrus reticulata Blanco* (Qing Pi), *Citrus reticulata Blanco* (Chen Pi), *Angelica sinensis (Oliv.) Diels* (Dang Gui), *Ligusticum chuanxiong Hort* (Chuan Xiong), *Pinellia ternata (Thunb.) Breit* (Ban Xia), *Fritillaria thunbergii Miq* (Zhe Bei Mu), *Forsythia suspensa (Thunb.) Vahl* (Lian Qiao), *Angelica pubescens Maxim. f. biserrata Shan et Yuan* (Du Huo) and *Glycyrrhiza uralensis Fisch* (Gan Cao). In China, several scholars have conducted studies demonstrating that HYD is effective in treating hyperthyroidism in clinical practice. Furthermore, certain animal experiments have indicated that HYD can notably improve hyperthyroidism. Evidence has validated the pharmacological role of HYD in inhibiting oxidative stress, and regulating cell proliferation, apoptosis, and cytokines (8). Nonetheless, no study has yet investigated the specific mechanism and targets of HYD for the treatment of hyperthyroidism. In the present study, we screened key targets of hyperthyroidism and active pharmaceutical ingredients (APIs) in HYD. Through network pharmacology and enrichment analyses, we illustrated hub genes of HYD in treating hyperthyroidism, and their enriched functions and signaling pathways. Network pharmacology, a bright guiding light on the way to explore the personalized precise medication of TCM, does not take into account the holistic and integrative efficacy of herbs (9). The clinical medication characteristics of TCM involve multiple components, targets, and signaling pathways of action, making it challenging to clarify the underlying mechanism using traditional methods. To establish its credibility, the results from network pharmacology need to be effectively combined with pharmacology and pharmacodynamic-related experiments. Together, network pharmacology provides a new opportunity for precisely treating both the manifestation and the root cause of disease. The interactions between APIs in HYD and therapeutic targets of hyperthyroidism were validated by molecular docking and non-targeted serum metabolomics. Through integrating network pharmacology, molecular docking, and non-targeted serum metabolomics, we clarified the pharmacodynamic material basis and pharmacologic mechanism of HYD in treating hyperthyroidism.

## Materials and methods

### Instruments

Ultrasonic cleaning machine (F-060SD, Shenzhen Fuyang, China), vortex oscillator (TYXH-I, Shanghai Hanuo Instruments, China), benchtop high-speed refrigerated centrifuge (TGL-16MS, Shanghai BioRidge, China), ACQUITY UPLC^®^ I-Class system (Waters Corporation, US), ACQUITY HSS T3 Columns (100 mm×2.1 mm, 1.8 µm, Waters Corporation), ACQUITY UPLC PDA Detector (Waters Corporation), and Q Exactive™ Plus Hybrid Quadrupole-Orbitrap™ Mass Spectrometer (Thermo Fisher Scientific, US) were used in the present study.

### Drugs and reagents

Traditional Chinese herbal medicines composed of HYD, including *Sargassum pallidum (Turn.) C. Ag* (Hai Zao), *Laminaria japonica Aresch* (Kun Bu), *Citrus reticulata Blanco* (Qing Pi), *Citrus reticulata Blanco* (Chen Pi), *Angelica sinensis (Oliv.) Diels* (Dang Gui), *Ligusticum chuanxiong Hort* (Chuan Xiong), *Pinellia ternata (Thunb.) Breit* (Ban Xia), *Fritillaria thunbergii Miq* (Zhe Bei Mu), *Forsythia suspensa (Thunb.) Vahl* (Lian Qiao), *Angelica pubescens Maxim. f. biserrata Shan et Yuan* (Du Huo) and *Glycyrrhiza uralensis Fisch* (Gan Cao) were provided by the Affiliated Hospital of Integrated Traditional Chinese and Western Medicine, Nanjing University of Chinese Medicine. Methanol (HPLC, A452-4), acetonitrile (HPLC, A998-4), and formic acid (HPLC, A117-50) were provided by Fisher Chemical, US. Distilled water was used in the experiments.

### Experimental animals

Six Sprague-Dawley (SD) rats weighing 200 ± 10 g in the specific pathogen-free (SPF) level were provided by SPFBiotech, Beijing, China (No. SCXK, Beijing, 2019-0010). They were housed in the Experimental Animal Center of the Affiliated Hospital of Integrated Traditional Chinese and Western Medicine, Nanjing University of Chinese Medicine (No. SYXK, Jiangsu, 2021-0025). Animal procedures were approved by the Ethics Committee of the Affiliated Hospital of Integrated Traditional Chinese and Western Medicine, Nanjing University of Chinese Medicine (No. AEWC-20221215-253).

### APIs in HYD

*Saccharina japonica* (Hai Dai) and *Laminaria japonica Aresch* (Kun Bu) are two sources described in the *Laminariales* in the *Pharmacopoeia of the People’s Republic of China 2020 Edition*. Therefore, *Saccharina japonica* (Hai Dai) and *Laminaria japonica Aresch* (Kun Bu) were considered as one herbal medicine in the present study. APIs in the 11 traditional Chinese herbal medicines of HYD were searched in the traditional Chinese medicine systems pharmacology (TCMSP, https://old.tcmsp-e.com/tcmsp.php), and those with oral bioavailability (OB) ≥30% and drug-likeness (DL) ≥0.18 were selected. Predicted API targets were then annotated using the Uniprot database (https://www.uniprot.org/).

Therapeutic targets of hyperthyroidism were obtained by intersecting those searched in the OMIM (https://www.omim.org/), DisGeNET (https://www.disgenet.org/), DrugBank (https://go.drugbank.com/), and GeneCards (https://www.genecards.org) databases. A Venn diagram was depicted to illustrate an intersection dataset involving APIs in HYD and key targets of hyperthyroidism using an online tool (http://www.bioinformatics.com.cn/). APIs in HYD and therapeutic targets of hyperthyroidism were imported into Cytoscape 3.7.2 to visualize and annotate a drug-ingredient-target network.

The overlapped targets of APIs in HYD and hyperthyroidism were imported into the STRING database (https://cn.string-db.org/), set at the Homo sapiens, and the highest confidence of 0.4. The hub genes were visualized in a PPI network using Cytoscape 3.7.2, and subjected to GO and KEGG enrichment analyses using the DAVID database (https://david.ncifcrf.gov/). Visualized in plots, the top 20 enriched signaling pathways in KEGG enrichment analysis and GO terms were yielded using Cytoscape 3.7.2.

Molecular docking is a computational tool that simulates the three-dimensional structure of molecules and intermolecular forces through physical chemometrics, identifies intermolecular interactions, and predicts the binding force and binding mode between molecules. It has been widely used in the design of drug structures and optimization of primers (10). We selected the top 10 hub genes in the network interacting APIs in HYD and therapeutic targets of hyperthyroidism and predicted their optimal ligands in the Protein Data Bank (PDB). The top 5 APIs with the highest degree of connectivity were imported into Autodock 4.2.6. Molecular docking results were visualized using the Autodock/Vina plugin for PyMOL.

### Drug preparation, animal drug administration and sample collection

On HYD formulation was composed of *Sargassum pallidum (Turn.) C. Ag* (Hai Zao) (12 g), *Laminaria japonica Aresc*h (Kun Bu) (18 g), *Citrus reticulata Blanco* (Qing Pi) (9 g), *Citrus reticulata Blanco* (Chen Pi) (9 g), *Angelica sinensis (Oliv.) Diels* (Dang Gui) (9 g), *Ligusticum chuanxiong Hort* (Chuan Xiong) (9 g), *Pinellia ternata (Thunb.) Breit* (Ban Xia) (9 g), *Fritillaria thunbergii Miq* (Zhe Bei Mu) (9 g), *Forsythia suspensa (Thunb.) Vahl* (Lian Qiao) (9 g), *Angelica pubescens Maxim. f. biserrata Shan et Yuan* (Du Huo) (9 g) and *Glycyrrhiza uralensis Fisch* (Gan Cao) (10 g). All traditional Chinese herbals were placed in a 5-liter round-bottom flask, and boiled in 10 times and 8 times amount of water for 1.5 h, respectively. Decoctions were mixed, concentrated into the crude drug at 2 g/mL, and placed at 4°C.

After habituation for 7 days, rats were randomly divided into a blank control group (n=3) and a HYD group (n=3) based on body weight. They were given free access to food and water. Rats in the blank control group were given oral gavage of normal saline, and those in the HYD group were given crude HYD extract at a dose of 22.96 g/kg in the same way. Calculated according to the equivalent dose in humans, rats were administered with 2.016 g/200 g HYD in a volume of 8 mL/kg twice a day, for 6 consecutive days.

Eating was avoided for 12 hours before blood sample collection. On day 7, rats were anesthetized by urethane (Shanghai Yuanye Biotechnology, China). Retro-orbital blood in rats was collected after anesthesia for 30 min, 60 min, 120 min, and 180 min, placed in anticoagulation tubes, and centrifuged at 3000 r/10 min within 30 min. The plasma sample of each rat was stored for later use.

### Non-targeted serum metabolomics

ACQUITY HSS T3 Columns (100 mm×2.1 mm, 1.8 um) were used in non-targeted serum metabolomics with formic acid 0.1% in mobile phase A and acetonitrile solution in mobile phase B. The gradient of the mobile phase was ramped as follows: 0-2 min, 5% B; 2-4 min, 5%-30% B; 4-8 min, 30%-50% B; 8-10 min, 50%-80% B; 10-15 min, 80%-100% B; 15-16 min, 5% B. Column temperature of 45°C, flow rate of 0.35 mL/min and injection volume of 5 μL of extract were prepared. The photodiode array (PDA) detection wavelength ranged from 210 nm to 400 nm.

Two separate chromatographic methods in both positive and negative modes were employed via a heated electrospray ionization (HESI) source using the following parameters: sheath gas, 35 arbitrary units (Arb); auxiliary gas, 8 Arb; capillary voltage, 3.8 kV for positive ion mode and 3.0 kV for negative ion mode; capillary temperature, 320°C; S-lens radio frequency level: 50; aux gas heater temperature, 350°C; data acquisition, Full MS/dd-MS2 Top 8; mass spectrum scanning range: *m/z* 100-1200; full scan resolution, 70,000; MS/MS scan resolution, 17500; collision energy, 10 eV, 20 eV and 40 eV.

Serum metabolic profiling was revealed via ultra-high-performance liquid chromatography with quadrupole time-of-flight mass spectrometry (UPLC-Q-TOF-MS). Liquid chromatography-mass spectrometry (LC-MS) raw data were processed for baseline filtering, peak identification, integration, retention time correction, peak alignment, and normalizations using the nonlinear, dynamic Progenesis QI v2.3 (Waters Corporation). The mass and measurement errors were below 5 ppm and 10 ppm, respectively. Serum metabolomics was characterized and annotated based on the accurate mass, secondary fragment structure, and isotope distribution using Animal_DB, HERB database, and self-built database.

## Results

### APIs in HYD of network pharmacology, overlapped targets of HYD and hyperthyroidism

**Figure 1:**
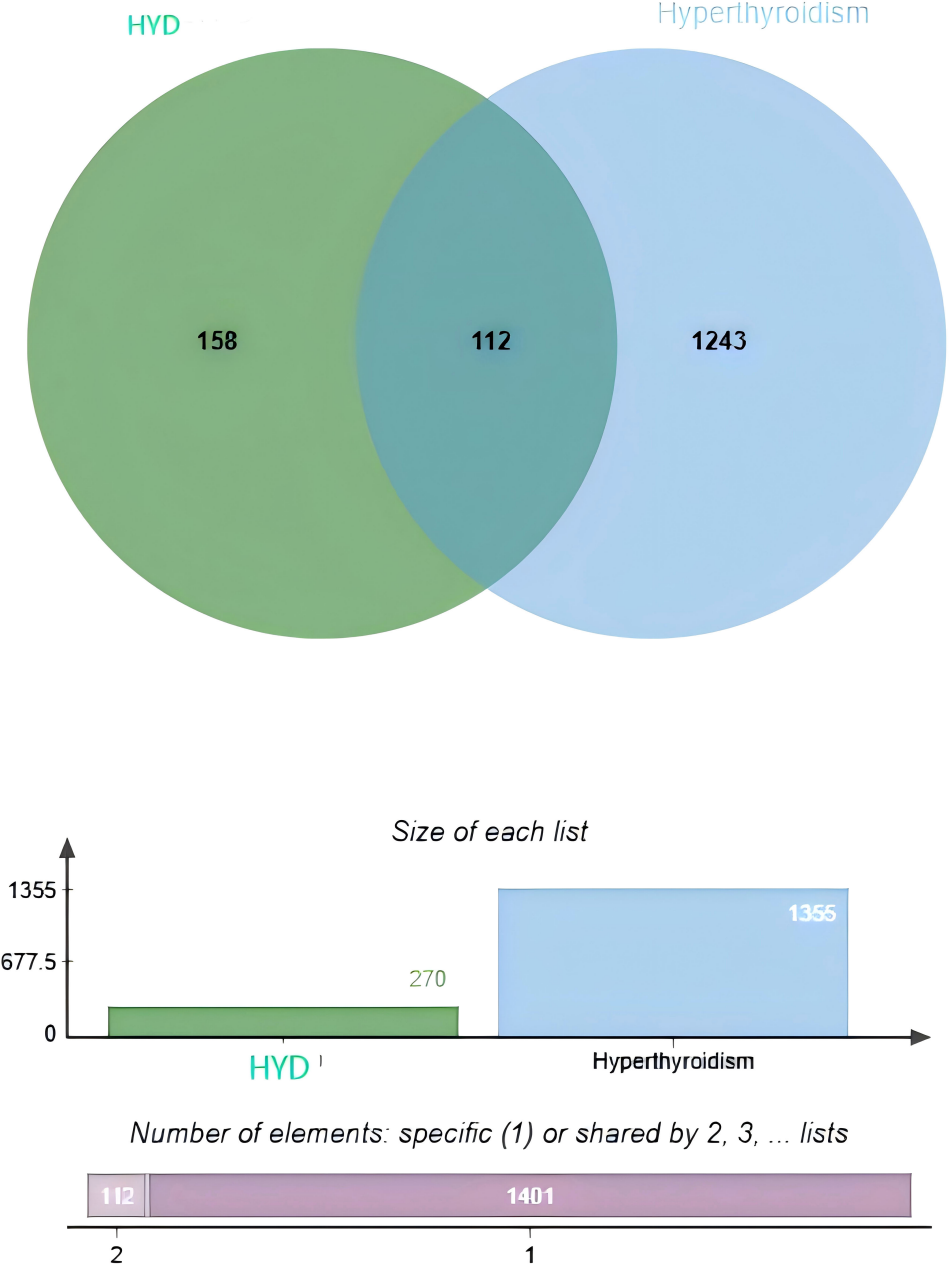
Venn diagrams visualizing an intersection dataset of active pharmaceutical ingredients in HYD and therapeutic targets of hyperthyroidism (112 common targets).

A total of 174 APIs in HYD, including repetitive ones, were identified. Specifically, there were 4, 7, 5, 5, 2, 7, 13, 7, 23, 9, and 92 APIs identified from *Sargassum pallidum (Turn.) C. Ag* (Hai Zao), *Laminaria japonica Aresch* (Kun Bu), *Citrus reticulata Blanco* (Qing Pi), *Citrus reticulata Blanco* (Chen Pi), *Angelica sinensis (Oliv.) Diels* (Dang Gui), *Ligusticum chuanxiong Hort* (Chuan Xiong), *Pinellia ternata (Thunb.) Breit* (Ban Xia), *Fritillaria thunbergii Miq* (Zhe Bei Mu), *Forsythia suspensa (Thunb.) Vahl* (Lian Qiao), *Angelica pubescens Maxim. f. biserrata Shan et Yuan* (Du Huo) and *Glycyrrhiza uralensis Fisch* (Gan Cao), respectively. A total of 1,355 targets of hyperthyroidism were predicted in the online databases. Finally, the Venn diagram identified 112 overlapped targets between HYD and hyperthyroidism (**Figure 1**).

### Drug-API-target network

**Figure 2:**
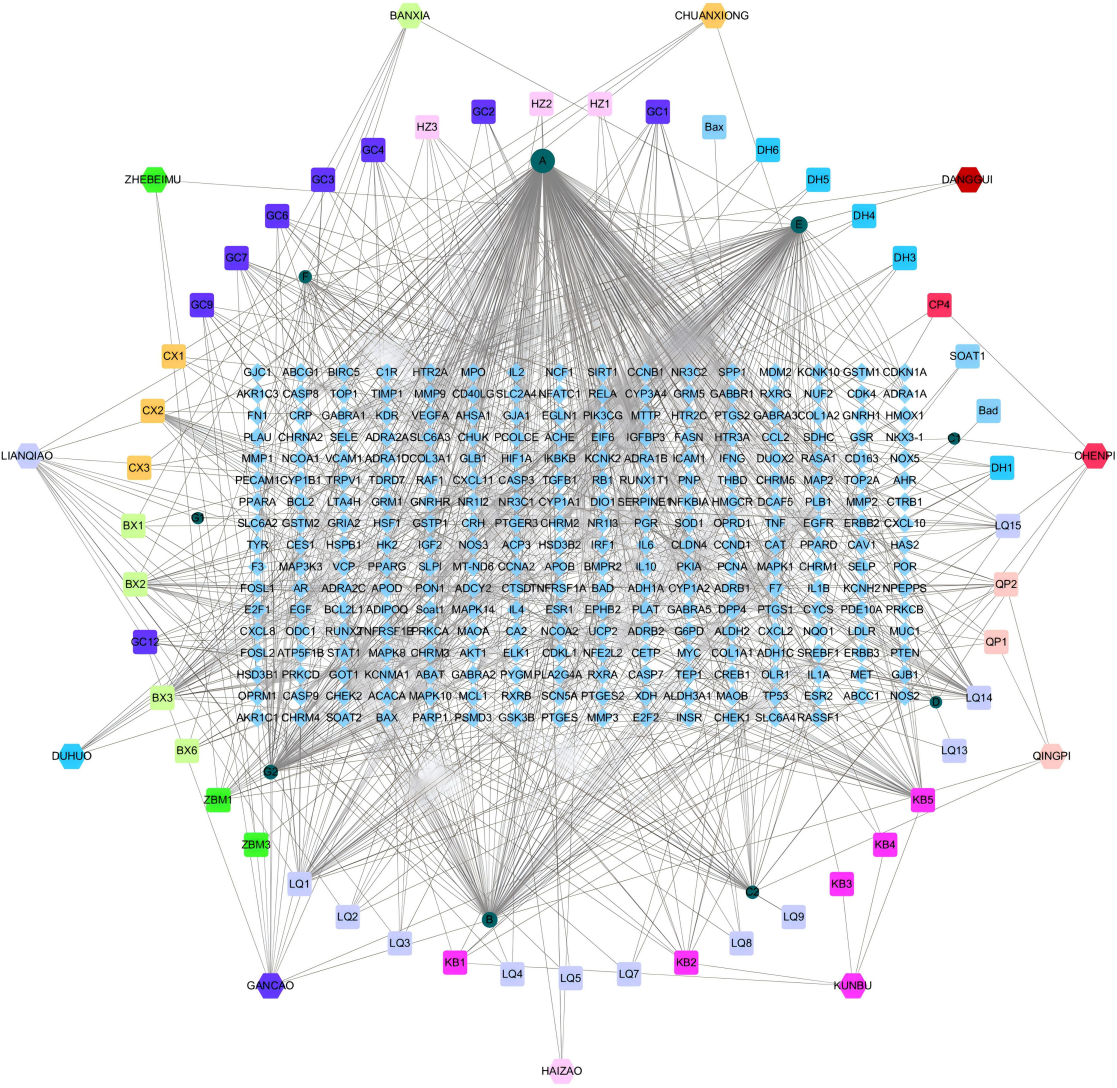
Network interaction diagrams visualizing the interactions of HYD, ingredients, and targets.

A drug-API-target network was constructed based on the overlapped targets, containing 337 nodes and 980 edges (**Figure 2**). The top 5 APIs based on the Degree value were selected as candidate ligands for molecular docking, involving quercetin (A, Degree=276), β-sitosterol (E, Degree=112), naringenin (B, Degree=83), kaempferol (G2, Degree=61) and wogonin (LQ1, Degree=38). In addition to wogonin, the remaining four ingredients repetitively appeared in the herbal medicines of HYD. The top 10 therapeutic targets in the drug-ingredient-target network were as follows: PPARG (Degree=14), NCOA2 (Degree=11), PTGS1 (Degree=11), PTGS2 (Degree=11), PGR (Degree=10), PIK3CG (Degree=10), SCN5A (Degree=10), BCL2 (Degree=10), CASP9 (Degree=10), and CASP3 (Degree=10).

### PPI, GO terms and KEGG signaling pathways

**Figure 3:**
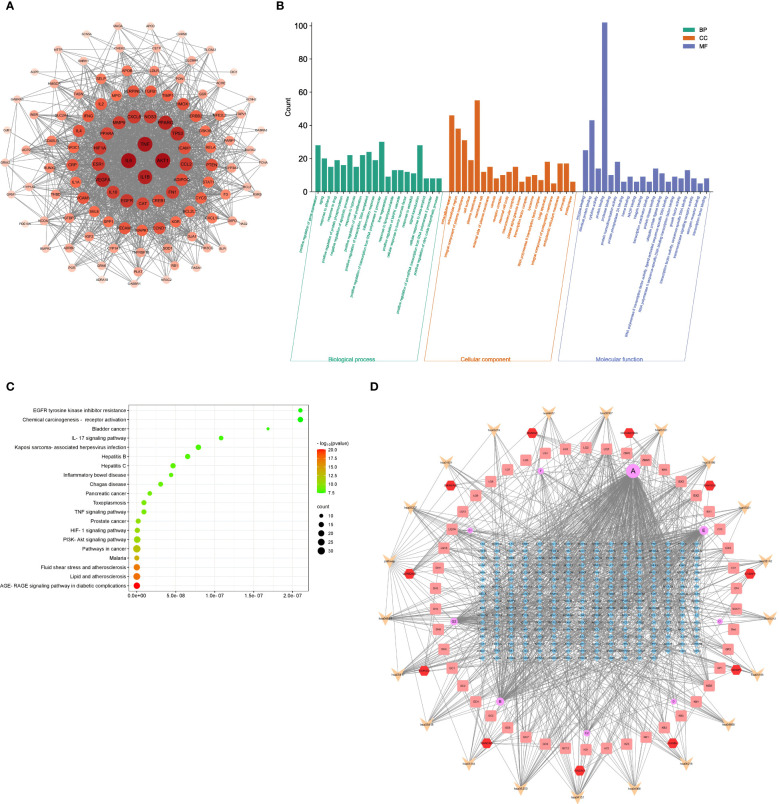
Results of PPI, GO terms, and KEGG signaling pathways. PPI networks visualizing the active pharmaceutical ingredients in HYD **(A)**; GO enrichment analysis of active pharmaceutical ingredients in HYD **(B)**; KEGG enrichment analysis of active pharmaceutical ingredients in HYD **(C)**; **(D)** Network interactions of HYD, ingredients, targets, and pathways. Node color denotes the p-value, and red and green represent less and greater significance, respectively. Node size denotes the number of enriched genes.

A PPI networks visualizing the active pharmaceutical ingredients in HYD (**Figure 3A**). Based on the Degree value, the top 10 genes were AKT1 (Degree=78), IL6 (Degree=78), TNF (Degree=76), IL1B (Degree=71), PPARG (Degree=69), VEGFA (Degree=68), TP53 (Degree=66), EGFR (Degree=61), CCL2 (Degree=60), and CXCL8 (Degree=60).

GO enrichment analysis was performed by importing 112 targets obtained in the drug-API-target network in the DAVID database. We screened 650, 110, and 76 terms of Biological Processes (BP), Molecular Function (MF), and Cellular Component (CC), respectively. The top 20 terms in each ontology were graphically visualized in **Figure 3B**.

A total of 148 signaling pathways were found enriched in the 112 targets using the DAVID database. The top 20 were visualized in a bubble chart (**Figure 3C**). Combining the top 20 signaling pathways and the drug-API-target network, we depicted a drug-API-target-pathway network (**Figure 3D**).

### Molecular docking results

**Figure 4:**
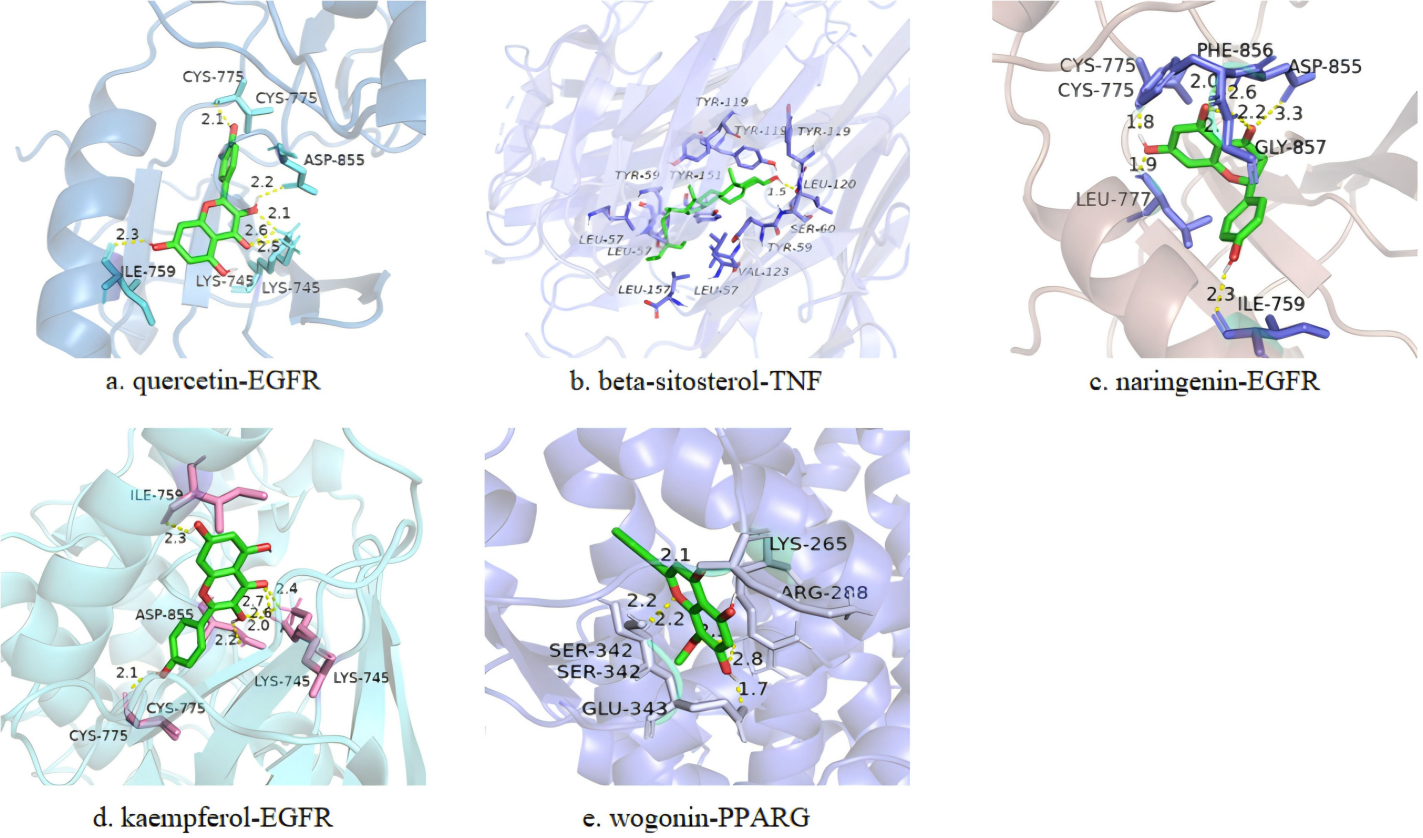
Representative docking pattens of the active ingredients in HYD with the strongest binding affinity to the key targets of hyperthyroidism. Binding affinity of quercetin-EGFR **(A)**, beta-sitosterol-TNF **(B)**, naringenin-EGFR **(C)**, kaempferol-EGFR **(D)** and wogonin-PPARG **(E)**.

Based on the PPI and drug-API-target network, the bindings of APIs of quercetin, β-sitosterol, naringenin, kaempferol and wogonin with key targets of IL6 (1alu), AKT1 (7nh5), TNF (5uui), PPARG (8b94), VEGFA (4kzn), TP53 (8dc8) and EGFR (8a27) were evaluated by molecular docking (**Table 1**). A binding force of lower than 0 kCal/mol indicates that an API can spontaneously bind to the target, and that of lower than -5 kCal/mol indicates a strong affinity. Those with the strongest binding affinity were visualized in **Figure 4**, where hydrogen bonds were labeled by dotted lines connecting atoms.

**Table 1:** Binding affinity (kCal/mol) of active ingredients of HYD to key targets of hyperthyroidism.

	Quercetin	β-sitosterol	Naringenin	Kaempferol	Wogonin
IL6 (1alu)	-5.75	-6.70	-6.16	-6.13	-6.14
AKT1 (7nh5)	-7.54	-7.90	-7.06	-7.7	-7.51
TNF (5uui)	-4.34	-12.30	-4.49	-4.52	-4.67
PPARG (8b94)	-6.5	-7.90	-6.26	-6.53	-8.31
VEGFA (4kzn)	-6.04	-6.30	-5.27	-5.85	-6.12
TP53 (8dc8)	-7.32	-6.50	-7.67	-7.66	-7.7
EGFR (8a27)	-8.37	-8.80	-7.89	-8.61	-7.62

### Non-targeted serum metabolomics analysis of absorbed compounds of HYD in plasma

**Figure 5:**
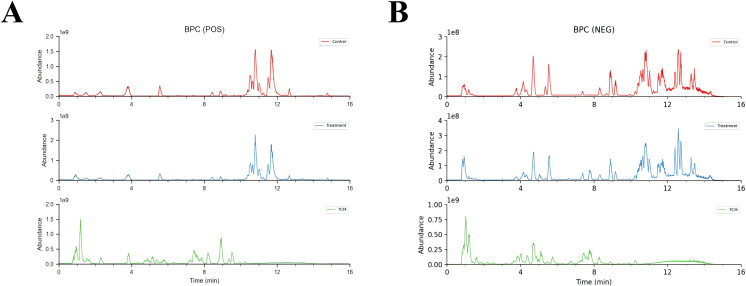
Base peak ion flow diagrams of UPLC-Q-TOF-MS/MS under positive **(A)** and negative ion modes **(B)**. Control means the intensity of mass spectrometry response of compounds in control blank serum; Treatment means the intensity of mass spectrometry response of compounds in serum treated with HYD; TCM means the intensity of mass spectrometry response of compounds in HYD original formulas.

The base peak ion flow diagrams of UPLC-Q-TOF-MS/MS under positive and negative ion modes were depicted in **Figure 5**. Based on the UPLC-Q-TOF-MS/MS fingerprinting data and literature review, a total of 279 absorbed compounds of HYD in plasma were identified by comparing compounds of known molecular weight and secondary fragment ions (**Supplementary Table 1**).

### Integration of network pharmacology and non-targeted serum metabolomics

**Figure 6:**
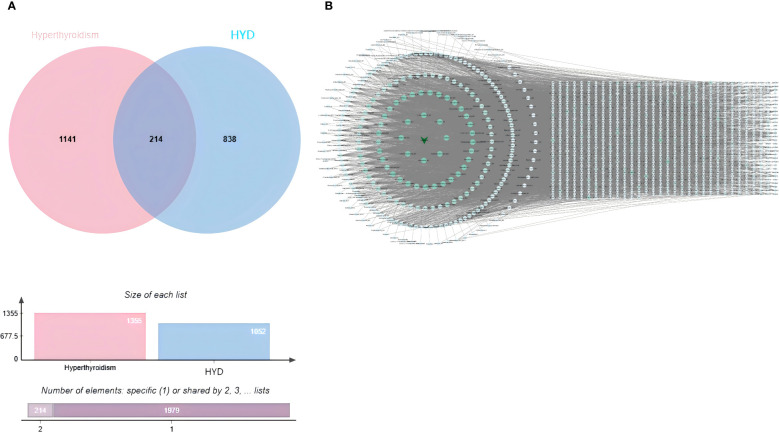
Venn diagrams visualizing an intersection dataset of absorbed compounds of HYD in plasma and therapeutic targets of hyperthyroidism (214 common targets in **A)**; **(B)** interactions of absorbed compounds of HYD in plasma and targets.

Compounds of HYD were predicted using the SwissTargetPrediction database. After excluding duplicates, a total of 1,052 targets of absorbed compounds of HYD in plasma with a probability > 0 points were identified. Through integrating 1,052 targets of compounds of HYD absorbed in the plasma and 1,355 targets of hyperthyroidism searched in public databases, 214 common targets were visualized in a Venn diagram (**Figure 6A**).

In the hexagonal map visualizing absorbed compounds of HYD in plasma (hexagons) and their targets (diamonds), the degree value of each node was calculated based on the size and color (**Figure 6B**). The top 10 nodes with the highest degree values were Sedanolide_M1, 11-Deoxyalisol B_M1, Tetrahydrocortisone_M1, Artemether_M1, Obacunone_M1, Osthole, Micromarin F, Columbianetin acetic acid, 3-Epioleanolic acid_M1 and Demethylsuberosin_M1.

### PPI network and GO and KEGG enrichment analyses of integration results

**Figure 7:**
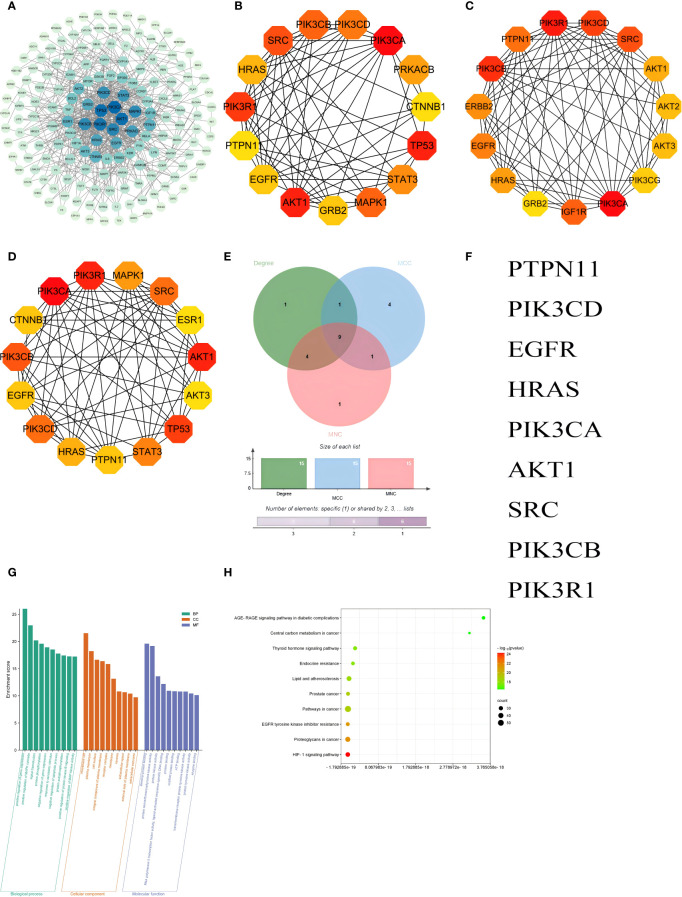
PPI network and GO and KEGG enrichment analyses of integration results. **(A)** PPI networks visualizing the absorbed compounds of HYD in plasma; The top 15 hub genes ranked based on the Degree **(B)**, Maximal Clique Centrality **(C)**, and Maximum Neighborhood Component **(D)**; A Venn diagram visualizing 9 hub genes in the intersection dataset of targets of absorbed compounds of HYD in plasma and those of hyperthyroidism **(E)** and detailed list **(F)**. GO enrichment analysis of absorbed compounds of HYD in plasma **(G)**, and KEGG enrichment analysis of absorbed compounds of HYD in plasma **(H)**. Node color denotes the p-value, and red and green represent less and greater significance, respectively. Node size denotes the number of enriched genes.

A protein-protein interaction (PPI) network involving 214 common targets was created using STRING and visualized in Cytoscape 3.7.2 (**Figure 7A**). The top 15 hub genes from the PPI network were selected by the algorithms of cytoHubba, including Degree, Maximal Clique Centrality (MCC), and Maximum Neighborhood Component (MNC) (**Figures 7B–D**). Finally, 9 hub genes were intersected with 214 common targets, including PTPN11, PIK3CD, EGFR, HRAS, PIK3CA, AKT1, SRC, PIK3CB and PIK3R1 (**Figures 7E, F**).

GO enrichment analysis revealed that the hub genes were mainly enriched in the BPs of positive regulation of gene expression, positive regulation of MAPK cascade, signal transduction, protein phosphorylation, negative regulation of gene expression, response to xenobiotic stimulus, negative regulation of apoptotic process, protein autophosphorylation, positive regulation of protein kinase B signaling and positive regulation of MAP kinase activity; CCs of membrane raft, plasma membrane, cell surface, integral component of plasma membrane and receptor complex; and MFs of identical protein binding, protein serine/threonine/tyrosine kinase activity, protein kinase activity, RNA polymerase II transcription factor activity, ligand-activated sequence-specific DNA binding and protein binding. The top 10 terms of BP, CC, and MF were visualized in **Figure 7G**. KEGG enrichment analysis predicted 185 signaling pathways enriched in the hub genes, including the HIF-1 signaling pathway, proteoglycans in cancer, EGFR tyrosine kinase inhibitor resistance, pathways in cancer, prostate cancer, lipid and atherosclerosis, endocrine resistance, thyroid hormone signaling pathway, central carbon metabolism in cancer and AGE-RAGE signaling pathway in diabetic complications and other pathways. The top 10 signaling pathways were visualized in **Figure 7H**.

## Discussion

Thyroid hormones are synthesized and secreted by thyroid follicular cells, which are responsible for regulating cell growth, proliferation, and metabolism. A bidirectional interaction exists between thyroid hormones and the immune system. Staphylococcus-stimulated lymphocytes contribute to synthesizing a TSH-like hormone that is critical in the pathogenesis of autoimmune thyroid diseases (11). Thyroid hormone levels elevate in hyperthyroidism patients, further inducing thyroid hyperplasia and even goiter by facilitating energy metabolism, cell proliferation, oxygen consumption, and protein phosphorylation. Generally, dysregulated angiogenesis, apoptosis, and proliferation are pathogenic factors of goiter. Proliferation of thyroid follicular cells and excessive growth of blood vessels, also known as pathological angiogenesis, are found in GD patients. The enlarged capillary lumen remarkably increases intrathyroid blood flow, leading to goiter or even refractory hyperthyroidism (12). The susceptibility to hyperthyroidism is also influenced by genetic polymorphisms of immune modulators, especially cytokines (13). Integrating network pharmacology, molecular docking, and non-targeted serum metabolomics, we illustrated pharmacodynamic APIs and the pharmacologic mechanism of HYD in treating hyperthyroidism. Therapeutic targets of hyperthyroidism were mainly involved in pathological angiogenesis, inflammatory response, cell apoptosis, and necrosis. Our findings heralded HYD as a promising prescription in the treatment of hyperthyroidism via inhibiting the pathological angiogenesis in the thyroid and rebalancing immunity.

Quercetin, β-sitosterol, naringenin, kaempferol and wogonin were screened out as major APIs in HYD. β-sitosterol influences angiogenesis by mediating the proliferation and migration of vascular smooth muscle cells via the PPARG/AMPK/mTOR signaling pathway (14). Moreover, β-sitosterol inhibits angiogenesis by inactivating the VEGF signaling pathway (15). Naringenin consistently inhibits angiogenesis via the VEGF signaling pathway (16). Both naringenin and kaempferol belong to the family of flavonoids, and the latter can regulate the migration and tube formation of endothelial cells by inactivating HIF-38α and VEGFR3 via the ERK/p38 MAPK and PI3K/Akt/mTOR signaling pathways. Kaempferol is found to inhibit microvascular sprouting in the mouse aortic ring assay (17). Serving as a competitive thyroid peroxidase (TPO) inhibitor, kaempferol exerts its therapeutic efficacy on hyperthyroidism by lowering the activity of TPO (18). Wogonin inhibits VEGF-mediated biological effects and the secretion of VEGF in tumor cells, thereby curbing angiogenesis (19). Major APIs in HYD can treat hyperthyroidism by inhibiting the pathological angiogenesis in the thyroid.

The immune imbalance of hyperthyroidism is often accompanied by a high production of cytokines. Major APIs in HYD also presented a potent anti-inflammation effect. To counter inflammation, quercetin relies on its abilities to suppress cyclooxygenase and lipoxygenase, maintain mast cell stability, and reduce the production of cytokines (e.g., TNF-α, IL-1β, IL-6). In addition to the inhibitory effects on anti-inflammatory factors, quercetin stimulates the release of pro-inflammatory cytokines like IL-10 (20). It also alleviates the inflammatory microenvironment by inhibiting the TLR/NF-κB and PI3K/AKT/NF-κB/STAT3 signaling pathways (21). Quercetin is believed as a promising compound for the treatment of thyroid ophthalmopathy by inhibiting IL-1β-induced active expressions of pro-inflammatory molecules, production of hyaluronic acid, and differentiation of adipocytes. The safety profile of quercetin is superior to that of high-dose glucocorticoids (22). Quercetin effectively protects thyroid cells against endocrine-disrupting chemicals by suppressing the activity of thyroid peroxidase and interfering with thyroid hormone metabolism (23, 24). Other APIs in HYD, like β-sitosterol, naringenin, kaempferol, and wogonin, play regulatory roles in the immune system as well (25–29). The diverse constituents within HYD operate synergistically in modulating cell proliferation, immune imbalance, anti-inflammation, and pathologic angiogenesis, thereby presenting a viable approach to the treatment of hyperthyroidism.

The KEGG enrichment analysis showed that the AGE-RAGE signaling pathway in diabetic complications, lipid and atherosclerosis, fluid shear stress and atherosclerosis, the PI3K-Akt signaling pathway, the HIF-1 signaling pathway, and the TNF signaling pathway were enriched in hub genes. Advanced glycation end-products (AGEs) polymers are formed by non-enzymatic reactions of proteins and lipids with carbohydrates. Through binding to the extensively expressed receptor for advanced glycation end-products (RAGE), the AGE-RAGE signaling pathway promotes inflammatory response, migration, invasion, and proliferation. AGEs participate in endothelial-mesenchymal transition (EMT), stimulate the production of reactive oxygen species (ROS) in vascular endothelial cells, enhance the vascular permeability, promote the migration of macrophages and T cells to the arterial intima, and upregulate VEGF to boost angiogenesis (30, 31). The PI3K-Akt signaling pathway is activated by dysfunctional receptor tyrosine kinases, and is responsible for mediating cell proliferation and angiogenesis (32). The hypoxia-inducible factor 1 (HIF-1) signaling pathway is involved in multiple biological processes, like cell survival, proliferation, angiogenesis, invasion, metastasis, and metabolic reprogramming (33).

Our bioinformatic analysis identified that the overlapped targets between HYD and hyperthyroidism were also important regulators of immunity. Interleukin-6 (IL-6) is an essential inflammatory cytokine that recruits and activates neutrophils. It is overexpressed during the aggravation of hyperthyroidism (34). IL-6 and tumor necrosis factor-α (TNF-α) levels are significantly higher in GD patients than in healthy volunteers, and they can be lowered by medication for ATDs (35). TNF is a vital cytokine involved in the immune response that directly activates inflammatory genes and indirectly accelerates inflammatory response and cell death (36). Meanwhile, TNF influences vasodilation and muscle capillary recruitment by activating the PI3K signaling pathway in endothelial cells (37). In addition to inflammatory factors, peroxisome proliferator-activated receptors (PPARs) also regulate inflammatory response and cell proliferation. The role of PPARs in endothelial cell homeostasis has been extensively analyzed. Loss of endothelial PPARγ greatly impedes angiogenesis (38).

Combining metabolomics and network pharmacology, an intersection dataset was fingerprinted, involving PTPN11, PIK3CD, EGFR, HRAS, PIK3CA, AKT1, SRC, PIK3CB, and PIK3R1 that were mainly enriched in the HIF-1 signaling pathway, EGFR tyrosine kinase inhibitor resistance, endocrine resistance, and thyroid hormone signaling pathway. They were responsible for the pharmacological mechanisms of HYD in treating hyperthyroidism.

The *PTPN11* gene encodes tyrosine phosphatase SHP2, a vital regulator of the MAPK signaling pathway. Dysregulation of SHP2 activates the downstream signals common in cell migration, differentiation, survival, and prognosis (39). PTPN11 prevents the differentiation of T cells into T helper 2 (TH2) cells (40). Through promoting the formation of neutrophil extracellular traps (NETs), PTPN11 aggravates inflammatory responses via releasing pro-inflammatory cytokines like TNF-α, IL-1β, IL-6, IL-17A and CXCL-15 (41). As an essential driving force of hyperthyroidism, inflammatory cytokines stimulate the aggregation and activation of neutrophils, influence vasodilation, and recruit blood capillaries by acting on the PI3K signaling pathway (34, 37). APIs in HYD are multifunctional to mediate cellular behaviors of the thyroid and inhibit the activation of inflammatory cytokines by targeting PTPN11. PIK3CA, PIK3CB and PIK3CD are the p110α, p110β and p110δ catalytic subunits of PI3K, respectively. Their activation poses a regulatory effect on cell proliferation by mediating downstream proteins of PI3K (42). PIK3R1 is the p85α regulatory subunit of PI3K that inhibits the catalytic activity of p110. Mutations or dysregulation of PIK3R1 alter metabolic functions by manipulating the activity of PI3K (43). EGFR is a transmembrane glycoprotein in the protein kinase superfamily. Serving as a receptor for members of the epidermal growth factor (EGF) family, EGFR stimulates cell proliferation by binding to epidermal growth factors and inducing receptor dimerization and tyrosine autophosphorylation (44). EGFR-induced complicated regulation of the ERK MAPK, PI3K-AKT, SRC, PLC-γ1-PKC, JNK, and JAK-STAT signaling pathway contributes to the activation of cell proliferation, growth, differentiation, migration, angiogenesis, and inhibition of cell apoptosis (45). Moreover, EGFR increases the activities of VEGF and VEGFR, it can also stimulate angiogenesis through upregulating hypoxia-dependent HIF-α. SRC is a non-receptor tyrosine-protein kinase that is closely linked with intracellular signal transductions, angiogenesis, metastasis and tumor progression (46). Activation of the SRC/STAT3 signaling pathway accelerates angiogenesis by upregulating pro-angiogenic molecules regulated by STAT3 transcription (47). HRAS is a GTPase in the RAS superfamily (48). Through acting on the RAS-RAF-MEK-ERK and RAS-PI3K-AKT-mTORC signaling pathways, HRAS provides instructions for regulating cell proliferation, migration, apoptosis, and survival (49). Targeting the effectors of the MEK/ERK and PI3K/δ/γ/Akt signaling pathways, RAS serves as a cellular switch to control angiogenesis and vascular permeability downstream of VEGF (50).

Targets of APIs in HYD were closely associated with signaling pathways for cell proliferation, angiogenesis, invasion, metastasis, metabolic reprogramming, endocrine resistance, and thyroid hormones. Multiple APIs in HYD contribute to the direct or indirect inhibition of endothelial cell migration, tubule formation, microvessel sprouting, and angiogenesis. Besides, they assist in rebalancing immunity by fighting against inflammation and oxidative stress. HYD employs multiple components, targets, and signaling pathways in treating hyperthyroidism, through inhibiting the pathogenic factors and alleviating clinical indicators. Integrating network pharmacology, molecular docking, and non-targeted serum metabolomics is an effective approach to offer a full-scale illustration of pharmacodynamic APIs and pharmacologic mechanisms. Our findings are of profound significance to guide the clinical management of hyperthyroidism using TCM prescriptions.

In our study, we used integrating network pharmacology, molecular docking, and non-targeted serum metabolomics to illustrate pharmacodynamic ingredients and pharmacologic mechanism of HYD in treating hyperthyroidism. It was also found that HYD may play a therapeutic role in treating hyperthyroidism by regulating immune imbalance and inhibiting pathologic angiogenesis, which closely matches the pathogenesis of hyperthyroidism, suggesting that HYD can be one of the therapeutic options for hyperthyroidism. And we had evaluated the reliability and validity of the findings by existing literature and clinical observations. Nevertheless, the study has some limitations that have to be acknowledged. We should admit the limitations of relying on computational predictions in this article. APIs in HYD and therapeutic targets of hyperthyroidism should be further validated in *in vitro* and *in vivo* studies. Additionally, indications of iodine-rich TCM for the treatment of hyperthyroidism need a clear illustration, and which type of hyperthyroidism will be more effective in clinical research. Finally, potential mechanisms underlying the pharmacologic effects of HYD in treating hyperthyroidism require an in-depth exploration.

## Conclusion

Through integrating network pharmacology, molecular docking, and non-targeted serum metabolomics, we illustrated that HYD copes with hyperthyroidism via regulating cell proliferation, migration, and survival, inhibiting pathological angiogenesis in the thyroid, suppressing pro-inflammatory cytokines and rebalancing the immunity. The results of this study will provide a new theoretical basis for the clinical treatment of hyperthyroidism.

## Data Availability

The original contributions presented in the study are included in the article/**Supplementary Material**. Further inquiries can be directed to the corresponding authors.
