# Supplementary material for: Integrating network pharmacology, molecular docking and non-targeted serum metabolomics to illustrate pharmacodynamic ingredients and pharmacologic mechanism of Haizao Yuhu Decoction in treating hyperthyroidism
Source: Front Endocrinol (Lausanne). 2024 Sep 25;15:1438821. doi: 10.3389/fendo.2024.1438821 (PMC11462413; doi:10.3389/fendo.2024.1438821)
Supplement: Supplementary file 1 [file Table1.docx]

Supplementary Table 1. Characterizations of absorbed compounds of HYD in plasma.

| **No.** | **tR/min** | **Metabolites** | **m/z** | **Formula** | **Adducts** | **Fragment Ions** |
| --- | --- | --- | --- | --- | --- | --- |
| 1 | 5.06 | (+)-Balanophonin_M2 | 557.163 | C26H30O12 | M+Na | 199.0214,229.2279, 381.1311, 557.1632 |
| 2 | 5.75 | (+)-Phylliroside | 579.2087 | C27H34O11 | M+FA-H | 356.1273,371.1506, 579.2079 |
| 3 | 6.21 | Medioresil | 389.1594 | C21H24O7 | M+H, M+Na | 300.0986,321.1116, 335.1259,341.1388, 353.1373, 359.0765, 359.1468, 371.1478, 388.1774, 389.1208 |
| 4 | 10.22 | Osthole | 245.1173 | C15H16O3 | M+H, M+Na, M+K | 131.049, 189.0545, 245.1172 |
| 5 | 5.37 | Oleuropein | 563.1759 | C25H32O13 | M+Na | 269.0806, 563.173 |
| 6 | 3.71 | Neochlorogenic acid | 353.0878 | C16H18O9 | M-H, 2M-H | 135.0444, 179.0346, 191.0555, 353.0878 |
| 7 | 9.56 | Natsudaidain | 419.1335 | C21H22O9 | M+H, M+Na | 361.0915, 389.0865, 404.1104, 419.1334 |
| 8 | 4.97 | Naringin | 581.1865 | C27H32O14 | M+H, M+Na, M+K | 129.0546, 147.0649, 153.0182, 195.0289, 273.0756, 315.0868, 383.1125, 401.1224, 419.1339, 435.1282 |
| 9 | 7.17 | Micromarin F | 283.0941 | C15H16O4 | M+H-H2O, M+Na | 131.0496, 159.044, 173.0597, 175.0391, 187.039, 189.0546, 201.0547, 243.1015 |
| 10 | 8.17 | Meranzin | 261.1122 | C15H16O4 | M+NH4, M+Na, M+H, M+H-H2O | 57.0702, 149.0234, 189.0546, 243.1016, 261.1122, 277.1782, 277.2157 |
| 11 | 6.2 | Medicarpin_M1 | 445.114 | C22H22O10 | M-H | 85.0285, 95.0127, 99.0077, 112.9843, 113.0232, 175.0240, 229.7480, 254.0590, 269.0816, 445.1145 |
| 12 | 6.04 | Osthole_M2 | 309.0438 | C14H14O6S | M-H | 229.0866, 309.0437 |
| 13 | 5.34 | Matairesinoside | 519.1873 | C26H32O11 | M-H | 83.0128, 357.1347, 519.1896 |
| 14 | 4.99 | Marmesinin | 409.1492 | C20H24O9 | M+H | 187.0389, 229.0859, 247.0964, 409.1391, 409.1469 |
| 15 | 4.79 | Liquiritigenin-7-o-apiosyl(1-2)-glucoside | 551.1759 | C26H30O13 | M+H | 137.0233, 257.0804 |
| 16 | 9.7 | Licoricone | 383.1489 | C22H22O6 | M+H, M+Na | 299.091, 327.0859, 383.1492 |
| 17 | 8.27 | Licorice-saponin H2 | 823.4112 | C42H62O16 | M+NH4, M+Na, M+H | 453.3365, 469.3323, 471.3473, 647.3795, 823.4119 |
| 18 | 7.29 | Licorice saponin G2 | 839.4059 | C42H62O17 | M+H, M+K, M+Na | 141.0182, 451.3206, 469.3311, 487.3415, 645.3639, 663.373, 839.4063 |
| 19 | 9.59 | Licoisoflavone A | 355.1176 | C20H18O6 | M+H | 123.0441, 151.039, 179.0338, 189.0908, 201.0908, 229.0856, 299.055, 299.0916, 337.1054, 355.1173 |
| 20 | 7.17 | Osthole_M1 | 297.0735 | C15H14O5 | M+H, M+Na | 169.1046, 187.0383, 189.0546, 211.1147, 229.0871, 229.1256, 230.0690, 257.0806, 257.1566, 275.0920 |
| 21 | 5.03 | Osthole_M3-2 | 325.0387 | C14H14O7S | M-H | 96.959, 188.0401, 244.0431, 245.0816, 256.0289, 325.0385 |
| 22 | 5.14 | Kakuol_M1 | 273.0077 | C10H10O7S | M-H | 158.0602, 193.05, 204.0662, 226.0485, 273.0078 |
| 23 | 5.61 | Quercetagetin 3,5,6,7,3',4'-hexamethyl ether_M1-2 | 565.1551 | C26H28O14 | M+H | 359.076, 374.0995, 389.1227, 565.1555 |
| 24 | 5.39 | Suspenoidside B | 567.1722 | C25H30O12 | M+FA-H | 61.9872, 163.0391, 358.1369, 521.1683, 566.2067 |
| 25 | 8.19 | Sinensetin | 373.1281 | C20H20O7 | M+Na, M+K, M+H | 395.1094 |
| 26 | 6.73 | Semilicoisoflavone B_M1-2 | 527.1195 | C26H24O12 | M-H | 59.0129, 85.0283, 99.0074, 113.0232, 175.0249, 229.6079, 351.0878, 527.12 |
| 27 | 9.28 | Semilicoisoflavone B | 353.102 | C20H16O6 | M+H, M+Na | 153.0182, 353.1015 |
| 28 | 8.93 | Scutellarein tetramethyl ether | 343.1175 | C19H18O6 | M+H | 282.0887, 313.0706, 343.1173 |
| 29 | 4.48 | Schaftoside | 565.1553 | C26H28O14 | M+H, M+Na | 415.0982, 427.1024, 433.1126, 445.1111, 481.1119, 499.1240, 511.1233, 529.1342, 547.1447, 565.1535 |
| 30 | 4.71 | Rutin | 611.1609 | C27H30O16 | M+H, M+Na | 71.0492, 85.0284, 303.0496, 465.1025 |
| 31 | 5.64 | Quercetagetin 3,5,6,7,3',4'-hexamethyl ether_M1-1 | 563.1407 | C26H28O14 | M-H | 228.1747, 231.7425, 256.9897, 300.9723, 342.0388, 357.0619, 372.0839, 387.1086, 426.9194, 563.1406 |
| 32 | 4.76 | Osthole_M3-1 | 325.0386 | C14H14O7S | M-H | 96.9591, 188.0095, 215.071, 227.951, 227.981, 245.0817, 325.0382 |
| 33 | 8.89 | Quercetagetin 3,5,6,7,3',4'-hexamethyl ether | 403.1386 | C21H22O8 | M+Na, M+K, M+H | 395.0734, 410.0973, 425.1207 |
| 34 | 5.86 | Poncirin | 639.1938 | C28H34O14 | M+FA-H, 2M-H, M-H | 285.0769, 593.1885 |
| 35 | 5.05 | Pinoresinol 4-O-beta-D-glucopyranoside | 565.1928 | C26H32O11 | M-H, M+FA-H | 136.0157, 151.0393, 357.1347, 519.1887 |
| 36 | 5.14 | Peucedanol | 263.0924 | C14H16O5 | M-H | 175.0394, 245.0815, 263.0925 |
| 37 | 5.2 | Peimine | 432.3473 | C27H45NO3 | M+H | 271.0605, 414.3365, 432.1346, 432.3468 |
| 38 | 4.66 | Paeonol_M1 | 230.9966 | C8H8O6S | M-H | 151.0392, 171.1017, 187.0053, 230.9966 |
| 39 | 4.43 | Oxyimperatorin_M1 | 296.9712 | C11H6O8S | M-H | 80.9642, 212.0014, 216.006, 217.0139, 296.0937, 296.9703 |
| 40 | 7.61 | Kaurenoic acid_M1-1 | 509.2391 | C26H38O10 | M-H | 59.2276, 85.0285, 91.3871, 113.0234, 175.0242, 235.2549, 283.2638, 333.207, 508.3421, 509.2396 |
| 41 | 5.82 | Isoverticine | 432.3472 | C27H45NO3 | M+H | 414.3365, 432.3469 |
| 42 | 4.3 | Vicenin 2 | 595.1658 | C27H30O15 | M+H, M+Na, M+H-H2O | 475.1230, 481.1128, 499.1239, 511.1236, 523.1222, 529.1357, 541.1343, 559.1448, 577.1555, 595.1652 |
| 43 | 6.13 | alpha-Asarone_M1 | 273.0439 | C11H14O6S | M-H | 193.0863, 228.0235, 228.0533, 273.0439 |
| 44 | 5.98 | Echinatin_M1 | 351.0544 | C16H16O7S | M-H | 135.044, 146.9627, 271.0977, 351.0545 |
| 45 | 5.86 | Didymin | 595.2021 | C28H34O14 | M+H, M+K, M+Na | 153.0182, 161.0597, 195.0288, 263.0546, 287.0912, 329.1017, 397.1278, 415.1387, 433.1487, 449.1440 |
| 46 | 4.65 | Daidzin | 417.1179 | C21H20O9 | M+H | 255.065, 417.1179 |
| 47 | 0.92 | D-altrofurano-heptulose-3 | 191.0554 | C7H14O7 | M-H2O-H | 85.0284, 87.0077, 111.0077, 129.0185, 191.02, 191.0554 |
| 48 | 8.79 | Columbianetin acetic acid | 311.0889 | C16H16O5 | M+NH4, M+Na | 241.9224, 259.2055, 287.1654, 287.2003, 288.9740, 289.1821, 289.2155, 305.1711, 305.2110, 305.2440 |
| 49 | 4.28 | 6-Hydroxycoumarin_M1 | 240.9809 | C9H6O6S | M-H | 161.0237, 240.9812 |
| 50 | 4.14 | Esculetin_M1 | 256.976 | C9H6O7S | M-H | 155.1067, 177.0187, 190.0508, 213.1501, 228.5646, 256.9762 |
| 51 | 8.19 | 6-Demethoxytangeretin | 343.1176 | C19H18O6 | M+H | 313.0692, 343.1169 |
| 52 | 9.93 | 5-O-Demethylnobiletin | 389.123 | C20H20O8 | M+Na, M+K, M+H | 396.0811, 411.1049 |
| 53 | 9.46 | 5-Desmethylsinensetin | 359.1124 | C19H18O7 | M+H, M+Na | 298.0833, 326.0782, 344.088, 359.112 |
| 54 | 5.88 | 3-N-butyl-4,5-dihydrophthalide_M1 | 383.1348 | C18H24O9 | M-H | 59.0127, 72.992, 103.0026, 113.0233, 131.034, 193.0347, 383.1349 |
| 55 | 4.59 | 3-Feruloylquinic acid | 367.1034 | C17H20O9 | M-H, 2M-H | 85.0285, 93.0337, 134.0364, 173.0449, 191.0555, 193.0501, 367.1034 |
| 56 | 9.36 | 3,5,6,7,8,3',4'-Heptemthoxyflavone | 433.1493 | C22H24O9 | M+H, M+K, M+Na | 403.1018, 433.1488 |
| 57 | 4.92 | Edpetiline | 592.3845 | C33H53NO8 | M+H | 574.3734, 592.3841 |
| 58 | 4.22 | Ethyl ferulic acid_M1 | 273.0074 | C10H10O7S | M-H | 96.9591, 134.0364, 149.06, 178.0264, 193.05, 273.0075 |
| 59 | 7.55 | Isosinensetin | 373.1282 | C20H20O7 | M+H, M+K, M+Na | 343.0812, 373.128 |
| 60 | 5.13 | Hesperetin 7-O-glucoside | 465.1392 | C22H24O11 | M+H | 153.0182, 177.0545, 303.0859 |
| 61 | 4.97 | Isorhoifolin | 579.171 | C27H30O14 | M+H | 129.0548, 153.0181, 271.0599, 273.0753, 383.1121, 401.1226, 419.1332, 433.1126, 435.1278, 579.1699 |
| 62 | 5.26 | Isoliquiritin apioside | 551.176 | C26H30O13 | M+H | 137.0233, 147.044, 257.0806, 419.1336 |
| 63 | 5.5 | Isoliquiritigenin_M1 | 433.1127 | C21H20O10 | M+H, M+Na | 137.0232, 147.044, 257.0805, 433.1117 |
| 64 | 7.38 | Isoliquiritigenin | 257.0807 | C15H12O4 | M+H | 137.0233, 147.0441, 257.0806 |
| 65 | 5.26 | Iristectorin B | 493.1341 | C23H24O12 | M+H | 316.0573, 331.0807, 493.1327 |
| 66 | 5.78 | Honokiol_M1 | 377.07 | C18H20O8S | M-H2O-H | 253.1235, 297.1134, 377.0703 |
| 67 | 5.12 | Hesperidin | 609.1828 | C28H34O15 | M-H, M+FA-H | 286.0488, 301.0717, 609.1827 |
| 68 | 6.21 | Heraclenol | 305.102 | C16H16O6 | M+H, M+Na | 203.0338, 305.1017 |
| 69 | 5.22 | Ferulic acid methyl ester_M1 | 287.0231 | C11H12O7S | M-H | 192.0422, 207.0659, 287.0234 |
| 70 | 7.75 | Glycyrrhizic acid | 823.4112 | C42H62O16 | M+H | 453.3363, 454.3395, 471.3471, 647.3783 |
| 71 | 9.07 | Glycycoumarin | 367.1186 | C21H20O6 | M-H | 139.0392, 297.041, 309.0395, 367.1195 |
| 72 | 9.18 | Glyasperin C | 357.1697 | C21H24O5 | M+H | 191.1067, 221.1174, 235.1324, 289.1062, 300.0937, 301.0696, 301.1061, 356.1913, 356.2750, 357.1672 |
| 73 | 4.7 | Forsythoside H | 647.1948 | C29H36O15 | M+Na, M+K, M+NH4 | 647.1945 |
| 74 | 5.06 | Ferulic acid methyl ester_M2 | 407.0949 | C17H20O10 | M+Na | 181.0097, 199.0213, 229.4537, 231.0627, 231.3698, 249.0728, 407.0921 |
| 75 | 9.52 | Tangeretin | 373.1281 | C20H20O7 | M+H, M+K, M+Na | 343.081, 358.1043, 373.1279 |
| 76 | 5.16 | Bergaptol_M1 | 280.9761 | C11H6O7S | M-H | 201.0187, 280.0584, 280.976 |
| 77 | 4.65 | Violanthin | 579.1708 | C27H30O14 | M+H | 405.0969, 423.1072, 441.1178, 457.1132, 459.1266, 507.1284, 525.1389, 543.1495, 561.1603, 579.1702 |
| 78 | 5.78 | isoshinanolone | 237.0766 | C11H12O3 | M+FA-H | 193.0864, 237.0766 |
| 79 | 9.29 | Khusilic acid | 263.1289 | C14H18O2 | M+FA-H | 219.1389, 245.1183, 263.1293 |
| 80 | 7.71 | Kaurenoic acid_M2 | 495.2602 | C26H40O9 | M-H |  |
| 81 | 7.92 | Kaurenoic acid_M1-3 | 533.2356 | C26H38O10 | M+Na |  |
| 82 | 7.91 | Kaurenoic acid_M1-2 | 509.2392 | C26H38O10 | M-H |  |
| 83 | 8.13 | Kaurenoic acid_M1-4 | 509.2392 | C26H38O10 | M-H |  |
| 84 | 4.75 | Kaempferol 3-neohesperidoside | 595.1658 | C27H30O15 | M+H |  |
| 85 | 6.59 | Isopropyl ferulate_M1 | 315.0545 | C13H16O7S | M-H |  |
| 86 | 4.96 | Isomaglone_M1 | 489.1404 | C24H26O11 | M-H |  |
| 87 | 4.79 | Isohemiphloin | 433.1139 | C21H22O10 | M-H |  |
| 88 | 4.34 | Isoferulic acid_M1 | 388.1237 | C16H18O10 | M+NH4 |  |
| 89 | 5.62 | Isoeugenol acetate_M1 | 243.033 | C10H12O5S | M-H |  |
| 90 | 10.4 | isocupressicacid | 365.2334 | C20H32O3 | M+FA-H, 2M-H |  |
| 91 | 4.98 | Isoacteoside | 669.2038 | C29H36O15 | M+FA-H |  |
| 92 | 4.35 | icariside f2 | 447.1509 | C18H26O10 | M-H, M+FA-H |  |
| 93 | 6.85 | Levomenol_M1 | 427.1952 | C21H32O9 | M-H |  |
| 94 | 9.29 | Licochalcone E | 383.1501 | C21H22O4 | M+FA-H |  |
| 95 | 4.91 | Hydrastine_M1 | 350.1045 | C20H19NO6 | M-H2O-H |  |
| 96 | 5.16 | Magnolol_M2 | 475.161 | C24H28O10 | M-H |  |
| 97 | 10.17 | Methyl cholate_M2 | 421.2949 | C25H42O6 | M+H-H2O |  |
| 98 | 8.88 | Methyl cholate_M1 | 407.2801 | C24H40O5 | M-H, M+FA-H, 2M-H |  |
| 99 | 6.41 | Medicagenic acid-3-O-beta-D-glucuronic acid-28-O-beta-D-glucopyranoside | 839.4084 | C42H64O17 | M-H |  |
| 100 | 5.11 | matteucinin | 656.2551 | C30H38O15 | M+NH4 |  |
| 101 | 4.86 | Magnolol_M1-2 | 317.1383 | C18H20O5 | M+H |  |
| 102 | 7.21 | Licoisoflavone A_M1 | 531.1501 | C26H26O12 | M+H |  |
| 103 | 4.86 | Magnolol_M1-1 | 299.1279 | C18H20O5 | M+H-H2O |  |
| 104 | 9.78 | Lupiwighteone | 337.1082 | C20H18O5 | M-H |  |
| 105 | 4.19 | Liquiritigenin 7,4'-diglucoside | 625.1779 | C27H32O14 | M-H, M+FA-H |  |
| 106 | 5.11 | limocitrin-3-glucoside | 509.1288 | C23H24O13 | M+H |  |
| 107 | 5.65 | Ligustilide_M1 | 331.0491 | C12H14O6S | M+FA-H |  |
| 108 | 8.93 | licoricesaponine c2 | 805.4029 | C42H62O15 | M-H |  |
| 109 | 3.89 | icariside D2 | 323.1101 | C14H20O7 | M+NH4, M+Na, M+H-H2O |  |
| 110 | 4.89 | Hispidol_M1 | 863.2049 | C21H20O10 | 2M-H |  |
| 111 | 8.02 | clusin | 383.1501 | C22H26O7 | M-H2O-H |  |
| 112 | 5.75 | Dehydrovomifoliol | 267.1237 | C13H18O3 | M+FA-H |  |
| 113 | 6.76 | Dihydrotanshinone I_M1 | 455.1314 | C24H24O10 | M+H-H2O |  |
| 114 | 4.96 | Dihydroresveratrol_M1 | 405.1191 | C20H22O9 | M-H |  |
| 115 | 6.63 | Dihydromollugin_M1 | 471.1263 | C22H24O10 | M+Na |  |
| 116 | 9.53 | Dihydrocucurbitacin B_M1 | 539.2999 | C32H46O8 | M-H2O-H |  |
| 117 | 5.78 | dictamdiol B | 301.1046 | C15H18O5 | M+H-H2O, M+Na, M+NH4 |  |
| 118 | 6.12 | Dianoside G | 953.4774 | C48H76O20 | M-H2O-H |  |
| 119 | 6.62 | Demethylsuberosin_M1 | 309.0438 | C14H14O6S | M-H |  |
| 120 | 8.41 | elephantin | 397.126 | C20H22O7 | M+H, M+Na |  |
| 121 | 7.12 | Cynatratoside A_M1 | 547.2526 | C28H38O8 | M+FA-H |  |
| 122 | 6.91 | Cynanchone A | 329.102 | C18H16O6 | M+H, M+Na |  |
| 123 | 5.32 | cucurbitoside b | 551.1774 | C26H32O13 | M-H |  |
| 124 | 4.17 | Cryptochlorogenic acid | 353.0878 | C16H18O9 | M-H, 2M-H |  |
| 125 | 3.99 | Coniferin | 387.1293 | C16H22O8 | M+FA-H |  |
| 126 | 4.08 | Coniferaldehyde_M1 | 259.0279 | C10H12O6S | M-H |  |
| 127 | 6.07 | Cnidilide_M1 | 385.1507 | C18H26O9 | M-H |  |
| 128 | 7.24 | Dipotassium glycyrrhizinate | 821.3956 | C42H60O16-2 | M+NH4, M+Na, M+H |  |
| 129 | 5.59 | Embinin qt | 505.1355 | C23H24O10 | M+FA-H |  |
| 130 | 3.87 | Forsythoside E | 485.1629 | C20H30O12 | M+Na, M+K, M+NH4, M+H |  |
| 131 | 8.29 | Guggulsterone_M1 | 395.1897 | C21H32O5S | M-H |  |
| 132 | 8.02 | gmelofuran | 291.1239 | C15H18O3 | M+FA-H |  |
| 133 | 4.94 | Glycycoumarin_M1 | 513.1371 | C26H26O12 | M+H-H2O |  |
| 134 | 6.5 | Glyasperin C_M1-2 | 555.184 | C27H32O11 | M+Na |  |
| 135 | 6.51 | Glyasperin C_M1-1 | 531.1872 | C27H32O11 | M-H |  |
| 136 | 4.49 | Genipin_M1 | 287.0229 | C11H14O8S | M-H2O-H |  |
| 137 | 4.49 | Forsythoside C | 639.1937 | C29H36O16 | M-H |  |
| 138 | 4.86 | Epiafzelechin | 257.0809 | C15H14O5 | M+H-H2O |  |
| 139 | 4.95 | Eugenetin_M1-2 | 435.0901 | C18H20O11 | M+Na |  |
| 140 | 4.98 | Eugenetin_M1-1 | 411.0932 | C18H20O11 | M-H |  |
| 141 | 7.09 | Ethynyl Estradiol_M1 | 483.2037 | C27H34O9 | M-H2O-H |  |
| 142 | 5.81 | Ethyl p-methoxycinnamic acid_M1 | 317.0335 | C11H12O6S | M+FA-H |  |
| 143 | 4.74 | Eriocitrin | 597.1814 | C27H32O15 | M+H |  |
| 144 | 7.66 | erianthridin | 271.0976 | C16H16O4 | M-H |  |
| 145 | 3.61 | Mussaenosidic acid | 375.1296 | C16H24O10 | M-H, M+FA-H, 2M-H |  |
| 146 | 4.17 | N-Feruloyloctopamine_M1 | 566.0977 | C24H27NO14S | M-H2O-H |  |
| 147 | 5.08 | Narcissin | 625.1769 | C28H32O16 | M+H |  |
| 148 | 9.38 | Sofalcone_M1 | 465.193 | C27H30O7 | M-H |  |
| 149 | 5.88 | stelmatotriterpenoside h | 823.413 | C42H66O17 | M-H2O-H |  |
| 150 | 6.42 | Sorbifolin | 599.1176 | C16H12O6 | 2M-H |  |
| 151 | 2.77 | Sophoridine | 249.1962 | C15H24N2O | M+H |  |
| 152 | 4.98 | Sophorabioside | 577.1566 | C27H30O14 | M-H |  |
| 153 | 8.13 | Sophoflavescenol | 367.1186 | C21H20O6 | M-H |  |
| 154 | 5.78 | Sinensetin_M1 | 437.0551 | C19H18O10S | M-H |  |
| 155 | 6.85 | Tetrahydrocortisone_M1 | 361.2023 | C21H30O5 | M-H |  |
| 156 | 6.65 | Shikonin_M1 | 447.1292 | C22H26O11 | M-H2O-H |  |
| 157 | 5.13 | Senkyunolide I_M1 | 423.1263 | C18H24O10 | M+Na |  |
| 158 | 6.69 | Semilicoisoflavone B_M1-1 | 551.1162 | C26H24O12 | M+Na |  |
| 159 | 6.23 | Sedanolide_M1 | 423.2754 | C12H20O3 | 2M-H |  |
| 160 | 3.67 | Secologanoside 7-methyl ester | 413.1053 | C16H22O11 | M+NH4, M+Na |  |
| 161 | 4.64 | Rutarin | 469.1349 | C20H24O10 | M+FA-H |  |
| 162 | 4.49 | Roseoside | 431.1923 | C19H30O8 | M-H, M+FA-H |  |
| 163 | 7.1 | terrestrosin e | 941.4739 | C45H74O19 | M+Na |  |
| 164 | 4.7 | Yibeissine | 444.3108 | C27H41NO4 | M+H |  |
| 165 | 4.48 | Zingerone_M1 | 275.0597 | C11H16O6S | M-H |  |
| 166 | 5.15 | zhebeininoside | 594.4002 | C33H55NO8 | M+H |  |
| 167 | 9.91 | Zederone | 245.1181 | C15H18O3 | M-H |  |
| 168 | 4.76 | Zearalenone_M1 | 493.1715 | C24H32O12 | M-H2O-H |  |
| 169 | 4.66 | Thymol_M1-1 | 245.0486 | C10H14O5S | M-H |  |
| 170 | 9.26 | turgeniifolin c | 367.1154 | C19H20O6 | M+Na, M+K |  |
| 171 | 9.36 | Triptonoterpene methyl ether_M1-2 | 691.4222 | C21H30O4 | 2M-H |  |
| 172 | 9 | Triptonoterpene methyl ether_M1-1 | 345.207 | C21H30O4 | M-H |  |
| 173 | 4.81 | Thymol_M1-2 | 245.0486 | C10H14O5S | M-H |  |
| 174 | 5.05 | Rhoifolin | 623.1628 | C27H30O14 | M+FA-H |  |
| 175 | 4.74 | Reynoutrin_M1 | 527.0499 | C21H20O14S | M-H |  |
| 176 | 9.56 | Obacunone | 455.2059 | C26H30O7 | M+H |  |
| 177 | 4 | Osmanthuside H | 455.1525 | C19H28O11 | M+NH4, M+Na |  |
| 178 | 4.62 | Orientin | 447.0934 | C21H20O11 | M-H |  |
| 179 | 5.58 | Ononin | 431.1337 | C22H22O9 | M+H, M+K, M+Na |  |
| 180 | 4.35 | Olivil 4'-O-glucoside | 561.1944 | C26H34O12 | M+H-H2O, M+NH4, M+Na |  |
| 181 | 12.1 | Oleanolic Acid_M1 | 535.31 | C30H48O6S | M-H |  |
| 182 | 8.64 | Oleanane-2H, +2O, 1COOH, O-HexA-HexA | 825.4263 | C42H64O16 | M+H, M+K, M+Na |  |
| 183 | 7.74 | Obacunone_M1 | 967.3428 | C26H28O9 | 2M-H |  |
| 184 | 7.99 | Obacunoic acid | 517.2079 | C26H32O8 | M+FA-H |  |
| 185 | 4.49 | Rehmapicroside | 345.1555 | C16H26O8 | M-H |  |
| 186 | 4.08 | o-[β-d-xylopyranosyl(1→6)β-d-gluco-pyranosyl] 7-hydroxycoumarin | 501.1252 | C20H24O12 | M-H, M+FA-H |  |
| 187 | 9.25 | Nonivamide_M1 | 310.2013 | C17H27NO4 | M+H |  |
| 188 | 4.82 | Neoliquiritin | 419.1336 | C21H22O9 | M+H, M+NH4, M+Na, M+K |  |
| 189 | 5.11 | Neohesperidin | 611.1969 | C28H34O15 | M+NH4, M+K, M+Na, M+H, M+H-H2O |  |
| 190 | 5.96 | Neoglycyrol_M1 | 559.1457 | C27H28O13 | M-H |  |
| 191 | 5.06 | Neodiosmin | 607.1676 | C28H32O15 | M-H |  |
| 192 | 4.38 | Narirutin 4'-glucoside | 787.231 | C33H42O19 | M+FA-H |  |
| 193 | 4.5 | patrinoside | 507.2083 | C21H34O11 | M+FA-H |  |
| 194 | 5.35 | Peiminine | 430.3316 | C27H43NO3 | M+H-H2O, M+H |  |
| 195 | 5.08 | Peimisine | 428.316 | C27H41NO3 | M+H |  |
| 196 | 6.41 | rehmapicrogenin | 367.2126 | C10H16O3 | 2M-H |  |
| 197 | 9.55 | Quillaic acid_M1 | 517.3166 | C30H46O7 | M-H |  |
| 198 | 5.03 | Quercetagetin 3,5,6,7,3',4'-hexamethyl ether_M2 | 549.125 | C25H26O14 | M-H |  |
| 199 | 4.29 | Puerarin apioside | 593.1516 | C26H28O13 | M+FA-H |  |
| 200 | 4.29 | plantasioside | 521.1304 | C23H24O11 | M+FA-H |  |
| 201 | 5.43 | Piperlonguminine_M1 | 438.1759 | C21H27NO9 | M+H |  |
| 202 | 9.43 | pinusolidicacid | 355.1879 | C20H28O4 | M+Na |  |
| 203 | 8 | Picfeltarraenin IV | 923.4665 | C47H72O18 | M-H |  |
| 204 | 7.84 | phenethyl,vanillate | 303.1226 | C17H18O5 | M+H |  |
| 205 | 5.12 | Peucedanol_M1 | 325.0928 | C14H16O6 | M+FA-H |  |
| 206 | 5.55 | Pentamethylquercetin_M1 | 533.1303 | C25H26O13 | M-H |  |
| 207 | 5.83 | Tetrahydrocortisone_M2 | 445.1903 | C21H34O8S | M-H |  |
| 208 | 3.91 | Cistanoside F | 487.1456 | C21H28O13 | M-H |  |
| 209 | 7.23 | Albanin A_M1 | 527.1195 | C26H26O13 | M-H2O-H |  |
| 210 | 8.76 | Araboglycyrrhizin | 777.4079 | C41H62O14 | M-H |  |
| 211 | 8.7 | Apioglycyrrhizin | 801.4034 | C41H62O14 | M+NH4, M+Na, M+H |  |
| 212 | 7.19 | Angelol B | 377.1594 | C20H24O7 | M+H, M+K, M+Na |  |
| 213 | 7.43 | Angelol A | 377.1593 | C20H24O7 | M+H, M+K, M+Na |  |
| 214 | 7.59 | Alloimperatorin_M1 | 349.0389 | C16H14O7S | M-H |  |
| 215 | 9.35 | Alloimperatorin | 271.0965 | C16H14O4 | M+H, M+Na |  |
| 216 | 6.43 | Acbglu | 579.1498 | C30H26O12 | M+H, M+Na, M+H-H2O |  |
| 217 | 8.2 | abrusoside c | 807.4186 | C42H64O15 | M-H |  |
| 218 | 6.99 | Abrisaponin 1 | 1015.4771 | C48H74O20 | M+FA-H |  |
| 219 | 5.06 | A-dihydroconiferyl ether | 401.1573 | C20H26O7 | M+Na |  |
| 220 | 6.37 | 8-Prenylnaringenin_M1 | 515.156 | C26H28O11 | M-H |  |
| 221 | 4.58 | 6-Hydroxycoumarin_M2 | 256.9761 | C9H6O7S | M-H |  |
| 222 | 4.59 | 6-Hydroxy-2-(2-phenylethyl)chromone_M1 | 519.0579 | C23H22O13S | M-H2O-H |  |
| 223 | 4.41 | 6'-o-β-d-glucopyranosylgentiopicroside | 541.153 | C22H30O14 | M+Na |  |
| 224 | 5.2 | Aristolochic acid D_M1 | 532.071 | C23H19NO14 | M-H |  |
| 225 | 8.74 | Artemether_M1 | 567.3179 | C15H24O5 | 2M-H |  |
| 226 | 9.78 | 5-Pentadecylresorcinol_M1 | 493.2812 | C27H44O9 | M-H2O-H |  |
| 227 | 12.47 | Calcifediol_M1 | 443.3168 | C27H42O2 | M+FA-H |  |
| 228 | 5.97 | Cirsiliol_M1 | 565.1202 | C24H24O13 | M+FA-H |  |
| 229 | 6.47 | (+)-Balanophonin | 355.1188 | C20H20O6 | M-H |  |
| 230 | 4.3 | Cis-Ferulic acid 4-O-beta-D-glucopyranoside | 379.1002 | C16H20O9 | M+NH4, M+Na |  |
| 231 | 5.19 | cichorioside B | 485.1668 | C21H28O10 | M+FA-H |  |
| 232 | 6.24 | Casticin_M1 | 551.1399 | C25H26O14 | M+H |  |
| 233 | 4.82 | Cantleyine | 206.0817 | C11H13NO3 | M-H |  |
| 234 | 5.07 | Calycosin_M1 | 461.1074 | C22H20O11 | M+H |  |
| 235 | 6.15 | Bruceine A_M1 | 553.1929 | C25H32O11 | M+FA-H |  |
| 236 | 6.8 | Artesunate_M1 | 441.1764 | C21H32O11 | M-H2O-H |  |
| 237 | 6.28 | borneol-2-o-β-d-glucopyranoside | 361.1869 | C16H28O6 | M+FA-H |  |
| 238 | 11.76 | beta-Elemonic acid_M1 | 471.3465 | C30H46O4 | M+H |  |
| 239 | 9.67 | beta-Elemonic acid_M2 | 531.3327 | C30H46O5 | M+FA-H |  |
| 240 | 4.02 | Bergenin | 327.072 | C14H16O9 | M-H |  |
| 241 | 4.53 | berchemol-4'-o-β-d-glucoside | 537.1985 | C26H34O12 | M-H |  |
| 242 | 5.94 | Batatasin III_M1 | 347.0559 | C15H16O6S | M+Na |  |
| 243 | 4.59 | Batatasin IV_M1 | 497.0759 | C21H24O13S | M-H2O-H |  |
| 244 | 4.61 | 6'-o-e-feruloylmonotropein | 549.1603 | C26H30O14 | M+H-H2O |  |
| 245 | 7.09 | Calendasaponin B | 953.4768 | C48H76O20 | M-H2O-H |  |
| 246 | 6.07 | 5-O-Demethylnobiletin_M1 | 453.05 | C19H18O11S | M-H |  |
| 247 | 5.75 | 2-(2-Phenylethyl)chromone_M1-1 | 471.1263 | C24H24O11 | M+H-H2O |  |
| 248 | 6.8 | 2,4-Dihydroxychalcone_M1 | 445.114 | C22H22O10 | M-H |  |
| 249 | 8.76 | 2'-Methoxykurarinone_M1 | 465.1929 | C27H30O7 | M-H |  |
| 250 | 5.1 | 2'-Hydroxy-4'-methylacetophenone_M1 | 349.0893 | C15H18O8 | M+Na |  |
| 251 | 5.05 | 16-hydroxytriptolide | 357.134 | C20H24O7 | M-H2O-H |  |
| 252 | 12.35 | 11-Deoxyalisol B_M1 | 469.3323 | C30H46O4 | M-H |  |
| 253 | 4.59 | (R,R)-Secoisolariciresinol diglucoside | 667.2617 | C32H46O16 | M-H2O-H |  |
| 254 | 5.94 | 2-[4,5-dimethoxy-2-[(2S,3R,4S,5S,6R)-3,4,5-trihydroxy-6-(hydroxymethyl)oxan-2-yl]oxyphenyl]-5-hydroxy-3,6,7-trimethoxychromen-4-one | 567.1707 | C26H30O14 | M+H, M+Na |  |
| 255 | 4.21 | (e)-2-hexenyl-α-l-arabinopyranosyl-(1→2)-β-d-glucopyranoside | 439.1822 | C17H30O10 | M+FA-H |  |
| 256 | 4.35 | (?)-olivil-4''-o-β-d-glucopyranoside | 583.2037 | C26H34O12 | M+FA-H |  |
| 257 | 10.58 | (25R)-12alpha-Hydroxyspirost-4-en-3-one_M1 | 427.2851 | C27H42O5 | M-H2O-H |  |
| 258 | 5.09 | (+)-Balanophonin_M1 | 483.0964 | C20H22O9S | M+FA-H |  |
| 259 | 4.86 | (1s,4s,5s,7r,10r)-10,11,14-trihydroxyguai-3-one 11-o-β-d-glucopyranoside | 471.2012 | C21H36O9 | M+K |  |
| 260 | 6.95 | 5-hydroxymatatabiether | 335.2229 | C10H16O2 | 2M-H |  |
| 261 | 4.51 | (1r,2r)-p-menth-4(5)-ene-1,2-diol 1-o-β-d-glu-copyranoside | 355.1729 | C16H28O7 | M+Na |  |
| 262 | 5.77 | 2-(2-Phenylethyl)chromone_M1-2 | 469.1116 | C24H24O11 | M-H2O-H |  |
| 263 | 6.98 | 4-(5-isopropoxy-2-Methyl-4-nitrophenyl)pyridine_M1 | 575.2166 | C15H16N2O4 | M-H, 2M-H |  |
| 264 | 10.21 | 3-Oxo-7-hydroxychol-4-enoic acid_M1 | 783.5794 | C24H40O4 | 2M-H |  |
| 265 | 4.07 | 4-Feruloylquinic acid_M1 | 447.0604 | C17H20O12S | M-H |  |
| 266 | 7.53 | 2-Hydroxy-6-ethyljuglone | 219.0653 | C12H10O4 | M+H |  |
| 267 | 4.61 | 4-Feruloylquinic acid | 369.1181 | C17H20O9 | M+H, M+Na |  |
| 268 | 5.13 | 4'-Methoxypuerarin | 413.1226 | C22H22O9 | M+H-H2O |  |
| 269 | 5.86 | 4''-hydroxyimperatorin 4''-o-β-d-glucopyra-noside | 449.1441 | C22H24O10 | M+H |  |
| 270 | 8.99 | 3beta-Hydroxy-5-cholenoic acid_M1 | 435.2751 | C24H38O4 | M+FA-H, 2M-H, M-H |  |
| 271 | 8.89 | 3-Oxocholic acid_M1 | 431.2767 | C24H40O5 | M+NH4, M+Na |  |
| 272 | 4.81 | 4-O-β-D-glucopyranosyloxybenzoic acid | 549.1616 | C26H32O14 | M-H2O-H, M+FA-H |  |
| 273 | 5.78 | 5-Desmethylsinensetin_M1 | 423.0396 | C18H16O10S | M-H |  |
| 274 | 13.42 | 3-Epioleanolic acid_M1 | 453.3373 | C30H46O3 | M-H |  |
| 275 | 6.69 | 3-[(2S,3R)-2-(4-hydroxy-3-methoxy-phenyl)-3-(hydroxymethyl)-7-methoxy-2,3-dihydrobenzofuran-5-yl]propyl acetate | 401.1605 | C22H26O7 | M-H |  |
| 276 | 9.13 | 20-Deoxyingenol | 331.1915 | C20H28O4 | M-H |  |
| 277 | 6.06 | 3,4-di-o-caffeoylquinic,acid | 543.151 | C27H28O12 | M-H |  |
| 278 | 4.45 | 3-O-p-coumaroylquinic acid | 339.1075 | C16H18O8 | M+H, M+Na |  |
| 279 | 11.18 | 23-Hydroxybetulinic acid_M1 | 469.3323 | C30H46O4 | M-H |  |
